# Supplementary material for: The impact of electronic health record discontinuity on prediction modeling
Source: PLoS One. 2023 Jul 6;18(7):e0287985. doi: 10.1371/journal.pone.0287985 (PMC10325091; doi:10.1371/journal.pone.0287985)
Supplement: S1 File — (DOCX) [file pone.0287985.s001.docx]

**Supplemental Materials**

**Supplemental Table 1: Inclusion Criteria and Covariate Definition (**please also see **Appendix 1.3**, **1.4, and 1.5** in the excel file for corresponding **ICD-10 codes):**

| **Variable** | **Definition** | **Comments** |
| --- | --- | --- |
| Coronary Artery Disease (CAD) | ICD-9 Dx: 410.x-414.x, 429.2  V45.81 |  |
| Systemic embolism | ICD-9: 444.xx (arterial embolism) |  |
| DVT | - - 1. ICD-9 : 451.xx, 453.xx |  |
| PE | ICD-9: 415.11, 415.12, 415.19 |  |
| Atrial fibrillation/flutter | one ICD-9 diagnosis code of 427.3 (Atrial fibrillation/flutter), 427.31 (atrial fibrillation), 427.32 (Atrial flutter) |  |
| Valvular disease | At least 1 inpatient or outpatient ICD-9 Dx code of 394.x – 397.x, 398.9x, V42.2 (heart valve replaced by transplant), V43.3 (heart valve replaced by a mechanical device/prosthesis)1 OR ICD-9 procedure code 35.1x (open heart valvuloplasty without replacement), 35.2x (replacement of heart valve)  OR  one of the following CPT codes:  33660-33665 (atrioventricular valve repair)  33400-33403 (aortic valve valvuloplasty)  33420-33430 (mitral valve repair/valvuloplasty/replacement)  33463-33468 (tricuspid valve repair/valvuloplasty/replacement)  33496 (prosthetic valve dysfunction repair) |  |
| Hypertension | At least 1 Dx of ICD-9 codes 401.x – 405.x  OR  At least 1 dispensing of a CCB, ACEI, ARB, BB, a thiazide diuretic or a direct antihypertensive agent |  |
| Diabetes | At least 2 outpatient diagnoses of DM (ICD-9 250.X (diabetes)) OR 1 hospital discharge Dx of DM OR 1 diagnosis of DM plus an insulin or oral antidiabetic dispensing |  |
| Hyperlipidemia | ICD-9 272.0, 272.2, 272.4 |  |
| Atherosclerosis | ICD-9 440.9 (arteriosclerosis)  ICD-9 414.X (other forms of chronic ischemic heart disease)  ICD-9 429.2 (ASCVD) |  |
| Heart failure (CHF) | 1 inpatient or 2 outpatient claims with any of ICD-9 codes : 428.x, 398.91, 402.01, 402.11, 402.91, 404.01, 404.11, 404.91, 404.03, 404.13, 404.93 | Validated in Medicare algorithm:  Hospital discharge ICD-9 codes: 428.x, 398.91, 402.01, 402.11, 402.91, 404.01, 404.11, 404.91, 404.03, 404.13, 404.93  PPV 0.97, Spec. 0.97, Sens. 0.76[^1^](#_ENREF_1) |
| Hemorrhagic stroke | 1 inpatient or 2 outpatient claims with any of 430.x – 432.x, |  |
| Ischemic stroke | 1 inpatient or 2 outpatient claims with any of ICD-9 codes: 433.x, 434.x, 436.x, 437.1 | Differs from the outcome definition as higher sensitivity is preferred for covariates definition |
| Other Stroke Effects | 1 inpatient or 2 outpatient claims with any of  ICD-9 codes: 438.x |  |
| Previous TIA | ICD-9 435.xx |  |
| COPD | 491.xx, 492.xx, or 496.xx |  |
| Asthma | 493.xx |  |
| Pneumonia | 480.xx – 486.xx, 487.0x, 507.xx |  |
| Alcohol abuse | Alcohol abuse ICD-9 codes:  94.61 – 94.63 – alcohol rehabilitation and detoxification  94.67-94.69 – combined alcohol/drug rehabilitaion and detoxification  303.0x – 303.9x – alcoholism  291.xx – alcohol-induced mental disorders  357.5x – alcoholic polyneuropathy  425.5x – alcoholic cardiomyopathy  571.1x – acute alcoholic hepatitis  571.2x – alcoholic cirrhosis of liver  571.3x – alcoholic liver damage, unspecified  305.0x -alcohol abuse |  |
| Drug abuse or dependence | 292.xx, 304.xx, 305.2x-305.9x |  |
| Epilepsy or convulsions | 345.xx, 780.3x |  |
| Cancer | 140.x-195.x, 196.x-198.x, 199.x, 200.x-208.x, 230.x-234.x, 235.x-238.x, 239.x, excluding non-melanoma skin cancer ( = 173.xx), v10.xx |  |
| Metastatic Cancer | 196, 196.0, 196.1, 196.2, 196.3, 196.5, 196.6, 196.8, 196.9, 197, 197.0, 197.1, 197.2, 197.3, 197.4, 197.5, 197.6, 197.7, 197.8, 198, 198.0, 198.1, 198.2, 198.3, 198.4, 198.5, 198.6, 198.7, 198.8, 198.81, 198.82, 198.89, 199, 199.0, 199.1, 199.2 |  |
| Lung Cancer | 162.2, 162.3, 162.4, 162.5, 162.8, 162.9, 231.2, V10.11 |  |
| Breast Cancer | 174.0, 174.1, 174.2, 174.3, 174.4, 174.5, 174.6, 174.8, 174.9, 175.0, 175.9, 233.0, V10.3 |  |
| Prostate Cancer | 185, 233.4, V10.46 |  |
| Melanoma | 172.0, 172.1, 172.2, 172.3, 172.4, 172.5, 172.6, 172.7, 172.8, 172.9, V10.82 |  |
| Stomach Cancer | 151.0, 151.1, 151.2, 151.3, 151.4, 151.5, 151.6, 151.8, 151.9, 230.2, V10.04 |  |
| Pancreatic Cancer | 157.0, 157.1, 157.2, 157.3, 157.4, 157.8, 157.9 |  |
| Colorectal Cancer | 153.0, 153.1, 153.2, 153.3, 153.4, 153.5, 153.6, 153.7, 153.8, 153.9, 159.0, 230.3, V10.05, 154.0, 154.1, 154.2, 154.3, 154.8, 230.4, 230.5, 230.6, V10.06 |  |
| Uterine Cancer | 179, 182.0, 182.1, 182.8, 233.2, V10.42 |  |
| Leukemia | 202.40, 202.41, 202.42, 202.43, 202.44, 202.45, 202.46, 202.47, 202.48, 203.1, 203.10, 203.11, 204.0, 204.00, 204.01, 204.1, 204.10, 204.11, 204.2, 204.20, 204.21, 204.8, 204.80, 204.81, 204.9, 204.90, 204.91, 205.0, 205.00, 205.01, 205.1, 205.10, 205.11, 205.2, 205.20, 205.21, 205.3, 205.30, 205.31, 205.8, 205.80, 205.81, 205.9, 205.90, 205.91, 206.0, 206.00, 206.01, 206.1, 206.10, 206.11, 206.2, 206.20, 206.21, 206.8, 206.80, 206.81, 206.9, 206.90, 206.91, 207.0, 207.00, 207.01, 207.1, 207.10, 207.11, 207.2, 207.20, 207.21, 207.8, 207.80, 207.81, 208.0, 208.00, 208.01, 208.1, 208.10, 208.11, 208.2, 208.20, 208.21, 208.8, 208.80, 208.81, 208.9, 208.90, 208.91, V10.60, V10.61, V10.62, V10.63, V10.69 |  |
| Non-Hodgkin Lymphoma | 200.00, 200.01, 200.02, 200.03, 200.04, 200.05, 200.06, 200.07, 200.08, 200.10, 200.11, 200.12, 200.13, 200.14, 200.15, 200.16, 200.17, 200.18, 200.20, 200.21, 200.22, 200.23, 200.24, 200.25, 200.26, 200.27, 200.28, 200.30, 200.31, 200.32, 200.33, 200.34, 200.35, 200.36, 200.37, 200.38, 200.40, 200.41, 200.42, 200.43, 200.44, 200.45, 200.46, 200.47, 200.48, 200.50, 200.51, 200.52, 200.53, 200.54, 200.55, 200.56, 200.57, 200.58, 200.60, 200.61, 200.62, 200.63, 200.64, 200.65, 200.66, 200.67, 200.68, 200.70, 200.71, 200.72, 200.73, 200.74, 200.75, 200.76, 200.77, 200.78, 200.80, 200.81, 200.82, 200.83, 200.84, 200.85, 200.86, 200.87, 200.88, 202.00, 202.01, 202.02, 202.03, 202.04, 202.05, 202.06, 202.07, 202.08, 202.10, 202.11, 202.12, 202.13, 202.14, 202.15, 202.16, 202.17, 202.18, 202.20, 202.21, 202.22, 202.23, 202.24, 202.25, 202.26, 202.27, 202.28, 202.70, 202.71, 202.72, 202.73, 202.74, 202.75, 202.76, 202.77, 202.78, 202.80, 202.81, 202.82, 202.83, 202.84, 202.85, 202.86, 202.87, 202.88, 202.90, 202.91, 202.92, 202.93, 202.94, 202.95, 202.96, 202.97, 202.98, V10.71, V10.79 |  |
| Multiple Myeloma | 203.0, 203.00, 203.01, 203.8, 203.80, 203.81 |  |
| Radiation Therapy | ICD-9 procedure: 92.20, 92.21, 92.22, 92.23, 92.24, 92.25, 92.26, 92.27, 92.28, 92.29, 92.41  HCPCS/CPT: 55859, 55860, 55862, 55865, 76960, 77261, 77262, 77263, 77280, 77285, 77290, 77295, 77299, 77300, 77305, 77310, 77315, 77321, 77326, 77327, 77328, 77331, 77332, 77333, 77334, 77336, 77370, 77399, 77401, 77402, 77403, 77404, 77406, 77407, 77408, 77409, 77411, 77412, 77413, 77414, 77416, 77417, 77419, 77420, 77425, 77430, 77431, 77432, 77470, 77499, 77750, 77761, 77762, 77763, 77776, 77777, 77778, 77781, 77782, 77783, 77784, 77789, 77790, 77799, 79200, 79300, 79400, 79420, 79440, 79900, 79999 |  |
| Chemotherapy | ICD-9 procedure: 00.10, 00.15, 17.70, 99.25, 99.28  or  HCPCS/CPT: J9000-J9999, 96400, 96405, 96406, 96408, 96410, 96412, 96414, 96420, 96422, 96423, 96425, 96440, 96445, 96450, 96520, 96530, 96542, 96545, 96549, 99555  Or  *See chemotherapy Medication Use table below* |  |
| Bone Marrow Transplant | ICD-9 diagnosis: 41.0, 41.00, 41.01, 41.02, 41.03, 41.04, 41.05, 41.06, 41.07, 41.08, 41.09  HCPCS/CPT: 38230, 38231, 38240, 38241, 38242 |  |
| Morbid obesity | 278.01 |  |
| Dementia | 290.xx, 294.xx, 330.xx, 331.xx |  |
| Depression | 293.83, 296.2x. 296.3x, 298.0x, 300.4x, 309.0x, 309.1x, 309.28, 311.xx |  |
| Bipolar disorder | 296.0x, 296.1x, 296.4x, 296.5x, 296.6x, 296.7x, 296.8x, 296.99 |  |
| Psychosis | 290.8x, 290.9x, 295.xx, 297.xx, 298.xx, 299.xx, 780.1x |  |
| Personality disorder | 301.xx |  |
| Adjustment disorder | 309.21-309.23, 309.29, 309.3x, 309.4x, 309.82, 309.83, 309.89, 309.9x |  |
| Anxiety disorders | 293.84 Anxiety disorder in conditions classified elsewhere  300.0x Anxiety states  300.2x Phobic disorders  300.3x Obsessive-compulsive disorders  309.24 Adjustment disorder with anxiety  308.0x Anxiety as acute reaction to exceptional stress  309.81 Posttraumatic stress disorder |  |
| Generalized anxiety disorder | 300.02 |  |
| Previous MI | ICD-9 412.x | Separate variables for recent MI (ICD-9 410) and old MI (ICD-9 412) |
| Acute MI | ICD-9 410 |  |
| Peptic Ulcer Disease | Diseases of esophagus: 530.1x – 530.4x, 530.8x, 530.9x  Gastric ulcer: 531.x  Duodenal ulcer: 532.x  Peptic ulcer: 533.x  Acute gastritis: 535.0x  Other specified gastritis: 535.4x  Unspecified gastritis and gastroduodenitis: 535.5x  Duodenitis: 535.6x |  |
| Upper GI bleed | ICD-9 diagnosis: 531.0x, 531.2x, 531.4x, 531.6x  532.0x, 532.2x, 532.4x, 532.6x, 533.0x, 533.2x, 533.4x, 533.6x,  534.0x, 534.2x, 534.4x, 534.6x, 578.0 OR  ICD-9 procedure code 44.43 OR  CPT code 43255 |  |
| Lower/ unspecified GI bleed | ICD-9 diagnoses: 562.02, 562.03, 562.12, 562.13, 568.81, 569.3, 569.83, 569.85, 569.86  578.1x, 578.9 |  |
| Urogenital bleed | ICD-9 diagnoses:  Hematuria: ICD-9 Dx: 599.7  Excessive/frequent menstruation: ICD-9 Dx 626.2x AND secondary diagnosis indicating acute bleeding: anemia (280.0, 285.1, 285.9), orthostasis (458.0), syncope (780.2) |  |
| Other bleeds | Hemathrosis: 719.1x  Hemopericardium: 423.0x  Hemoptysis: 786.3x  Epistaxis: 784.7x  Hemorrhage not specified: 459.0x  Acute posthemorrhagic anemia: 285.1 |  |
| Peripheral Vascular disease or PVD surgery | 1 inpatient or 2 outpatient claims with any of the following codes:  ICD9 diagnosis:  440.20 - 440.24, 440.29 – 440.32, 440.3, 443.9    ICD9 procedure:  38.08, 38.09, 38.18, 38.48, 38.49, 39.25, 39.5, 39.9, 84.10 - 84.17  HCPCs:  35256, 35286, 35351, 35355, 35361, 35363, 35371, 35372, 35381, 35454, 35456, 35459, 35470, 35473, 35474, 35482, 35483, 35485, 35492, 35493, 35495, 35521, 35533, 35541, 35546, 35548, 35549, 35551, 35556, 35558, 35563, 35565, 35566, 35571, 35621, 35623, 35641, 35646, 35647, 35650, 35651, 35654, 35656, 35661, 35663, 35666, 35671, 27590, 27591, 27592, 27594, 27596, 27880, 27881, 27882, 27884, 27886, 27888 |  |
| Prior liver disease | ICD-9 diagnosis:  070.x viral hepatitis  571.x chronic liver disease and cirrhosis  572.x liver abscess and sequalae of chronic liver disease  573.x other disorders of liver  456.0 – 456.2x esophageal varices  155.0 primary cancer of liver  155.1 cancer of intrahepatic bile ducts  155.2 cancer of liver not specified as primary or secondary  576.8 cholestasis  ICD-9 procedure codes:  39.1 intra-abdominal venous shunt  42.91 ligation of esophageal varices |  |
| Renal Dysfunction | Acute renal disease (see below)  Chronic renal disease (see below)  Diabetic nephropathy (see below)  Hypertensive nephropathy (see below)  Miscellaneous Renal Insufficiency (see below)  Or ESRD (see below) | This definition of renal dysfunction is based on prior work[^3^](#_ENREF_3)^,^[^4^](#_ENREF_4) and will be used for covariate adjustment. It is a combination of several diagnoses that are not well distinguished in claims databases. |
| *Acute Renal Disease* | 580.0, 580.4, 580.8, 580.9, 581.0, 581.1, 581.2, 581.2, 581.3, 581.8, 581.9, 584.6, 584.7, 584.8, 584.9 |  |
| *Chronic Renal Insufficiency* | 582.x, 583.x, 585.x, 586.x, 587.x |  |
| *Diabetic Nephropathy* | 250.4, 250.40, 250.41,  250.42, 250.43 |  |
| *Hypertensive Nephropathy* | 403.xx, 404.xx |  |
| *Miscellaneous Renal Insufficiency* | 274.10, 440.1, 442.1, 453.3,  581.xx, 593.xx, 753.0,  753.3, 866.00 866.01,  866.1 |  |
| *ESRD (with and without dialysis)* | DIALYSIS  ICD-9 procedure:  39.95 hemodialysis  54.98 peritoneal dialysis  38.95 Venous catheterization for renal dialysis  39.27 Arteriovenostomy for renal dialysis  39.42 Revision of arteriovenous shunt for renal dialysis  39.43 Removal of arteriovenous shunt for renal dialysis  ICD-9 diagnoses:  V45.1 renal dialysis status  V56.0 extracorporeal dialysis  V56.8 peritoneal dialysis  CPT4:  90935      HEMODIALYSIS PROC W/SINGLE PHYSICIAN EVALUATION  90937      HEMODIALYSIS, REPEATED EVAL, W/WO REVISION DIALYSIS PRESCRIPTION  90940      HEMODIALYSIS ACCESS FLOW STUDY, BY INDICATOR DILUTION METHOD, HOOK UP;  MEASUREMENT & DISCONNECTION  90945      DIALYSIS, OTHER THAN HEMODIALYSIS, SINGLE PHYSICIAN EVAL  90947      DIALYSIS PROCEDURE, OTHER THAN HEMODIALYSIS, REPEATED PHYSICIAN EVAL  90989      DIALYSIS TRAINING, PATIENT, W/HELPER WHERE APPLICABLE, ANY MODE, COMPLETED COURSE  90993      DIALYSIS TRAINING, PATIENT, W/HELPER WHERE APPLICABLE, ANY MODE, COURSE INCOMPLETE, PER SESSION  99512      HOME VISIT, HEMODIALYSIS  99559      HOME INFUSION, PERITONEAL DIALYSIS, PER VISIT  OR  RENAL TRANSPLANT  V42.0    Kidney transplant  55.6x     Kidney transplant  996.81  Complication of transplanted kidney  CPT4:  50360       RENAL ALLOTRANSPLANTATION, IMPLANTATION, GRAFT; W/O DONOR & RECIPIENT NEPHRECTOMY  50365      RENAL ALLOTRANSPLANTATION, IMPLANTATION, GRAFT; W/RECIPIENT NEPHRECTOMY  50380      RENAL AUTOTRANSPLANTATION, REIMPLANTATION, KIDNEY  OR  RENAL ICD9-defined ESRD  585.5 ESKD with no mention of dialysis  585.6 ESKD on dialysis |  |
| Obesity | 278.00  278.01  V85.3x  V85.4x  CPT codes  '43842', '43843', '43846', '43847', '43848', 'G0443', 'G0447’  Prescriptions of orlistat, sibutramine, phentermine, benzphetamine, phendimetrazine, diethylpropion |  |
| Sepsis/Septicemia | 995.91, 995.92, 038.xx |  |
| Osteoarthritis | 715.xx |  |
| Rheumatoid arthritis | 714.0x, 714.1x, 714.2x |  |
| Neuropathic pain | 053.1x, 337.1x, 337.2x, 250.6x, 357.2, 350.1x, 350.2x, 352.1x, 353.xx, 354.xx, 355.xx, 357.xx, 729.2x, 721.1x, 721.41, 721.42, 721.91, 722.7x, 723.4x, 724.3x, 724.4 x |  |
| Back and neck pain without neuropathic involvement | 720.0x, 720.1x, 720.2x, 721.3x, 722.10, 722.32, 722.5x, 722.83, 722.93, 724.00, 724.02, 724.2x, 724.5x, 724.6x, 724.70, 724.71, 724.79, 720.81, 720.89, 720.9x, 721.0x, 721.2x, 721.5x, 721.6x, 721.7x, 721.8x, 721.90, 722.11, 722.30, 722.31, 722.39, 722.4x, 722.6x, 722.80, 722.81, 722.82, 722.90, 722.91, 722.92, 723.xx (except 723.4x), 724.01, 724.1x, 724.8x, 724.9x |  |
| Other arthritis, arthropathies and musculoskeletal pain | 710.xx−714.xx (excluding 714.0x-2x), 716.xx−719.xx, 725.xx−729.xx (excluding 729.1x-2x)] |  |
| Osteoporosis | 733.0 |  |
| Fibromyalgia | 729.1x |  |
| Migraine | 346.xx |  |
| Headache | 339.xx, 784.0x, 307.81 |  |
| Pain not elsewhere classified | 338.xx |  |
| Generalized pain | 780.96 |  |
| Pain disorders related to psychological factors | 307.8x (excluding 307.81) |  |
| Fractures | 733.1x, 800.xx-829.xx |  |
| Falls | E885, E885.9x, E888.xx |  |
| Coagulation disorders and purpura | 269.0x Vitamin K deficiency  286.xx Coagulation defects  287.xx Purpura and other hemorrhagic conditions  289.81 Protein S deficiency, protein C deficiency  289.82 Secondary hypercoagulable state  289.84 Heparin-induced thrombocytopenia (HIT) |  |
| Diseases of white blood cells | 288.xx |  |
| **Parkinson’s disease** | **332.xx, 333.0x** |  |
| Charlson comorbidity score | As a continuous variable and as a binary variable with levels: <3 vs >=3 |  |
| Anemia | 280.xx Iron deficiency anemia  281.xx Other deficiency anemias  282.xx Hereditary hemolytic anemias  283.xx Acquired hemolytic anemias  284.xx Aplastic anemia and other bone marrow failure syndromes  285.xx Other and unspecified anemias |  |
| HIV or AIDS | ICD-9-CM codes 042, 079.53, 795.71, V08 | [PLoS One.](http://www-ncbi-nlm-nih-gov.ezp-prod1.hul.harvard.edu/pubmed/26661399) 2015 Dec 14;10(12):e0144965 |
| Urinary incontinence | 788.3x, 788.91 |  |
| Decubitus ulcer | 707.0x |  |
| Oxygen | V46.2x |  |
| **Use of preventive services** | | |
| Mammography | CPT:  76082  76083  76092  HCPS:  G0202  G0203 |  |
| Pap test | HCPCS:  Q0091  P3000  P3001  G0123  G0124  G0141  G0143  G0145  G0147  G0148    CPT:  88141 – 88158  88164 – 88167  88174 – 88175 |  |
| PSA test | HCPCS:  G0103  CPT:  84153, 84152 – 84154 |  |
| Colonoscopy | 45.23  HCPCS:  G0105  G0121  CPT:  45378-45392 |  |
| Fecal occult blood test | HCPCS:  G0107  G0328  CPT:  82270  82274 |  |
| Flu shot | CPT:  90655 – 90660  90724  HCPCS: G0008  ICD-9 diagnosis:  V04.8  V04.81  V06.6 |  |
| Pneumococcal vaccine | 90669  90670  90732  V03.82 |  |
| BMD testing | CPT:  76070  76075  76076  76977 |  |
| **Medication use** |  |  |
| Any Antibiotic Use | Any dispensing of an antibiotic (see [appendix 1.2](#OLE_LINK4) for an antibiotics list), |  |
| Use of oral corticosteroids | See [appendix 1.1](#OLE_LINK4) for medication list |  |
| Aspirin/dipyridamole | any aspirin use in the baseline period |  |
| Aspirin monotherapy | Defined as any aspirin use in the baseline period without any dispensing of other antiplatelet agents (dipyridamole, Clopidogrel, Prasugrel, Cilostazol, ticlopidine, or Ticagrelor). |  |
| Clopidogrel monotherapy | Defined as any Clopidogrel use in the baseline period without any dispensing of other antiplatelet agents (dipyridamole, aspirin, Prasugrel, Cilostazol, ticlopidine, or Ticagrelor) |  |
| Aspirin and Clopidogrel dual therapy | Dispensing of both Aspirin and Clopidogrel in the baseline assessment period |  |
| Prasugrel | Prasugrel |  |
| Ticagrelor | Ticagrelor |  |
| Other antiplatelet agents | Cilostazol, ticlopidine |  |
| NSAIDs | See [appendix 1.1](#OLE_LINK4)for medication list |  |
| COX-2 inhibitors | See [appendix 1.1](#OLE_LINK4)for medication list |  |
| Opioids | See [appendix 1.1](#OLE_LINK4) for medication list |  |
| Diabetes medications | See [appendix 1.1](#OLE_LINK4)for medication list |  |
| Antidepressants | See [appendix 1.1](#OLE_LINK4)for medication list |  |
| Antipsychotics | See [appendix 1.1](#OLE_LINK4)for medication list |  |
| Anticonvulsants | See [appendix 1.1](#OLE_LINK4)for medication list |  |
| Proton pump inhibitor | See [appendix 1.1](#OLE_LINK4)for medication list |  |
| H2 receptor antagonist | See [appendix 1.1](#OLE_LINK4)for medication list |  |
| Other gastroprotective agents | Misoprostol, sucralfate |  |
| ACE-Inhibitors | See [appendix 1.1](#OLE_LINK4)for medication list |  |
| ARBs | See [appendix 1.1](#OLE_LINK4)for medication list |  |
| Beta-blockers | See [appendix 1.1](#OLE_LINK4)for medication list |  |
| Calcium channel blockers | See [appendix 1.1](#OLE_LINK4)for medication list |  |
| Thiazide diuretics | See [appendix 1.1](#OLE_LINK4)for medication list |  |
| Loop diuretics | See [appendix 1.1](#OLE_LINK4)for medication list |  |
| Potassium-sparing  diuretics | See [appendix 1.1](#OLE_LINK4)for medication list |  |
| Nitrates | See [appendix 1.1](#OLE_LINK4)for medication list |  |
| Aliskiren | See [appendix 1.1](#OLE_LINK4)for medication list |  |
| Other antihypertensives | See [appendix 1.1](#OLE_LINK4)for medication list |  |
| Antiarrhythmics | See [appendix 1.1](#OLE_LINK4)for medication list |  |
| Other anticoagulants | See [appendix 1.1](#OLE_LINK4)for medication list |  |
| Insulin | See [appendix 1.1](#OLE_LINK4)for medication list |  |
| Non-insulin hypoglycemics | See [appendix 1.1](#OLE_LINK4)for medication list |  |
| Digoxin | See [appendix 1.1](#OLE_LINK4)for medication list |  |
| Statins | See [appendix 1.1](#OLE_LINK4)for medication list |  |
| Other lipid-lowering agents | See [appendix 1.1](#OLE_LINK4)for medication list |  |
| SSRI |  |  |
| SNRIs | See [appendix 1.1](#OLE_LINK4)for medication list |  |
| TCAs | See [appendix 1.1](#OLE_LINK4)for medication list |  |
| MAOs | See [appendix 1.1](#OLE_LINK4)for medication list |  |
| Other newer and atypical antidepressants | See [appendix 1.1](#OLE_LINK4)for medication list |  |
| Lithium | See [appendix 1.1](#OLE_LINK4)for medication list |  |
| Atypical antipsychotics | See [appendix 1.1](#OLE_LINK4)for medication list |  |
| Typical antipsychotics | See [appendix 1.1](#OLE_LINK4)for medication list |  |
| Anticonvulsants | See [appendix 1.1](#OLE_LINK4)for medication list |  |
| Benzodiazepines | See [appendix 1.1](#OLE_LINK4)for medication list |  |
| Z-drugs | See [appendix 1.1](#OLE_LINK4)for medication list |  |
| Other anxiolytics/hypnotics | See [appendix 1.1](#OLE_LINK4)for medication list |  |
| Barbiturates | See [appendix 1.1](#OLE_LINK4)for medication list |  |
| Agents for dementia | See [appendix 1.1](#OLE_LINK4)for medication list |  |
| Hormone replacement therapies | See [appendix 1.1](#OLE_LINK4)for medication list |  |
| Osteoporosis | See [appendix 1.1](#OLE_LINK4)for medication list |  |
| Vitamin K therapy | Vitamin K |  |
| Cardiovascular Medications for Inclusion Criteria | Composite of above definitions for:  Aspirin/dipyridamole, aspirin monotherapy, Clopidogrel, Prasugrel, Cilostazol, Ticlopidine, Ticagrelor, Other Antiplatelet, ACE-Inhibitors, ARBs, Beta-blockers, Calcium channel blocker, Thiazide diuretics, Loop diuretics, Potassium-sparing diuretics, Nitrates, Aliskiren, Other antihypertensives, Antiarrhythmics, Insulin, Non-insulin hypoglycemics, Digoxin, Statins, Other lipid-lowering agents, and Diabetes medications |  |
| Number of Medications | Number of distinct generic names |  |
| Number of Hospitalizations |  |  |
| Number of hospital days |  |  |
| Number of Office visits |  |  |
| Number of cardiologist visits |  |  |
| Number of neurologist visits |  |  |
| Hospitalization in 30 days prior to index date |  |  |
| Number of laboratory tests ordered |  |  |
| Number of lipid tests ordered |  |  |

**Supplemental Table 2. Validated model to calculate the predicted EHR-continuity**

| **Variable** | **Coefficient** |
| --- | --- |
| Intercept | -0.010 |
| Having seen the same provider twice | 0.049 |
| Having seen the same provider >=3 times | 0.087 |
| Having general medical exam* | 0.078 |
| Mammography* | 0.075 |
| Pap smear* | 0.009 |
| PSA Test* | 0.103 |
| Colonoscopy* | 0.064 |
| Fecal occult blood test* | 0.034 |
| Influenza vaccine* | 0.102 |
| Pneumococcal vaccine* | 0.031 |
| Having BMI recorded* | 0.017 |
| Having 2 of the above routine care facts** | 0.049 |
| With any one medication use record | 0.002 |
| With at least 2 medication use records | 0.074 |
| Having A1C ordered or value recorded* | 0.018 |
| Having at least one inpatient or outpatient encounter | 0.091 |
| Having at least two outpatient encounters | 0.050 |
| With 1 diagnosis recorded in the EHR | -0.026 |
| With at least 2 diagnoses recorded in the EHR | 0.037 |
| Having any ED visit in the EHR | 0.078 |
| ** having 2 of the facts followed by *, EHR=electronic health records, PSA= prostate specific antigen | |

AUC for predicting the measured encounter capture proportion of ≥ 60% (a cut-off informed by literature^1^) was 0.86 in the training and 0.88 in the testing set.

The predicted EHR-continuity value was highly correlated with the measured EHR-continuity both in the training and testing set (Spearman coefficient =0.78 and 0.82, respectively)^2^

**Supplemental Table 3. Definition of the predictor for continuity**

| **Variable** | **Definition** |
| --- | --- |
| Having general medical exam or routine care* | ICD-9: V70.0 or V72.3 |
| Mammography* | ICD-9: V76.12 ICD-10: Z1231  CPT: 76082 76083 76092  HCPCS: G0202 G0203 |
| Pap smear* | HCPCS: Q0091 P3000 P3001 G0123 G0124 G0141 G0143 G0145 G0147 G0148  CPT: 88141 – 88158 88164 – 88167  88174 – 88175 |
| PSA Test* | HCPCS: G0103  CPT: 84153, 84152 – 84154 |
| Colonoscopy* | ICD-9: 45.23; ICD-10: 0DJD8ZZ  HCPCS: G0105 G0121  CPT: 45378-45392 |
| Fecal occult blood test* | HCPCS: G0107 G0328  CPT:82270 82274 |
| Influenza vaccine* | CPT: 90655 – 90660 90724  HCPCS: G0008  ICD-9: V04.8 V04.81 V06.6 |
| Pneumococcal vaccine* | CPT: 90669 90670 90732  ICD-9: V03.82 ; ICD-10: Z23 |
| Having BMI recorded* | With BMI recorded in the EHR |
| Having A1C ordered or value recorded* | Ordering or with results available in the EHR for HbA1c |
| Having 2 of the above routine care facts** | As described |
| With any one medication use record | As described |
| With at least 2 medication use records | As described |
| With 1 diagnosis recorded in the EHR | As described |
| With at least 2 diagnoses recorded in the EHR | As described |
| Having at least one inpatient or outpatient encounter in EHR | As described |
| Having at least two outpatient encounters in the EHR | As described |
| Having ED visit in the EHR | As described |
| Having seen the same provider twice | As described |
| Having seen the same provider >=3 times | As described |
| ** having 2 of the facts followed by *  BMI: body mass index; CPT: Current Procedural Terminology; ED: emergency department; HCPCS: Healthcare Common Procedure Coding System; ICD-9: International Classification of Diseases, Ninth Revision; EHR=electronic health records, PSA= prostate specific antigen | |

**Supplemental Table 4: Outcome variable definitions (**please also see **Appendix 1.3** excel file for corresponding **ICD-10 codes):**

| **Outcome** | **Hospital Discharge Code(s)** | **Comments** |
| --- | --- | --- |
| **Major cardiovascular events** | **A composite outcome of myocardial infarction, ischemic stroke, hemorrhagic stroke, other stroke effects, DVT, PE, or heart failure:** |  |
| Acute Myocardial infarction (MI) | ICD-9 Dx 410.X (acute myocardial infarction) |  |
| Hemorrhagic stroke | 1 inpatient or 2 outpatient claims with any of 430.x – 432.x, |  |
| Ischemic stroke | 1 inpatient or 2 outpatient claims with any of ICD-9 codes: 433.x, 434.x, 436.x, 437.1 |  |
| Other Stroke Effects | 1 inpatient or 2 outpatient claims with any of  ICD-9 codes: 438.x |  |
| DVT | ICD-9 : 451.xx, 453.xx |  |
| PE | ICD-9: 415.11, 415.12, 415.19 |  |
| Heart failure (CHF) | 1 inpatient or 2 outpatient claims with any of ICD-9 codes : 428.x, 398.91, 402.01, 402.11, 402.91, 404.01, 404.11, 404.91, 404.03, 404.13, 404.93 | Validated in Medicare algorithm:  Hospital discharge ICD-9 codes: 428.x, 398.91, 402.01, 402.11, 402.91, 404.01, 404.11, 404.91, 404.03, 404.13, 404.93  PPV 0.97, Spec. 0.97, Sens. 0.76[^1^](#_ENREF_1) |
| **Major hemorrhage** | **A composite outcome of upper GI bleed, lower GI bleed, urogenital bleed, or other bleed** |  |
| Upper GI bleed | ICD-9 diagnosis: 531.0x, 531.2x, 531.4x, 531.6x  532.0x, 532.2x, 532.4x, 532.6x, 533.0x, 533.2x, 533.4x, 533.6x,  534.0x, 534.2x, 534.4x, 534.6x, 578.0 OR  ICD-9 procedure code 44.43 OR  CPT code 43255 |  |
| Lower/ unspecified GI bleed | ICD-9 diagnoses: 562.02, 562.03, 562.12, 562.13, 568.81, 569.3, 569.83, 569.85, 569.86  578.1x, 578.9 |  |
| Urogenital bleed | ICD-9 diagnoses:  Hematuria: ICD-9 Dx: 599.7  Excessive/frequent menstruation: ICD-9 Dx 626.2x AND secondary diagnosis indicating acute bleeding: anemia (280.0, 285.1, 285.9), orthostasis (458.0), syncope (780.2) |  |
| Other bleeds | Hemathrosis: 719.1x  Hemopericardium: 423.0x  Hemoptysis: 786.3x  Epistaxis: 784.7x  Hemorrhage not specified: 459.0x  Acute posthemorrhagic anemia: 285.1 |  |

**Supplemental Table 5. Patient Characteristics of the study population in the training cohort**

| **Characteristics** | **Patients with high (top 20%) EHR- continuity**  **N = 138,294** | **Patients with low (lower 80%) EHR- continuity**  **N = 42,656** | **Standardized difference** |
| --- | --- | --- | --- |
| Age, years | 74.34 (7.68) | 76.05 (8.21) | -0.22 |
| Female | 60129 (43.5) | 17574 (41.2) | 0.05 |
| Race |  |  |  |
| White | 115364 (83.4) | 36023 (84.5) | -0.03 |
| Black | 3383 (2.4) | 1172 (2.7) | -0.02 |
| Asian | 2728 (2.0) | 822 (1.9) | 0.01 |
| North American Native | 101 (0.1) | 51 (0.1) | 0.00 |
| Unknown | 16718 (12.1) | 4588 (10.8) | 0.04 |
| **Medical history** |  |  |  |
| Coronary Artery Disease | 40045 (29.0) | 13500 (31.6) | -0.06 |
| Atrial Fibrillation | 24226 (17.5) | 8447 (19.8) | -0.06 |
| Hypertension | 123053 (89.0) | 38676 (90.7) | -0.06 |
| Heart Failure | 13230 (9.6) | 4864 (11.4) | -0.06 |
| Ischemic Stroke | 10272 (7.4) | 3348 (7.8) | -0.02 |
| Acute Myocardial Infarction | 4598 (3.3) | 1421 (3.3) | 0.00 |
| COPD | 19580 (14.2) | 6940 (16.3) | -0.06 |
| Pneumonia | 11979 (8.7) | 4583 (10.7) | -0.07 |
| Dementia | 13687 (9.9) | 6573 (15.4) | -0.17 |
| Depression | 27267 (19.7) | 9033 (21.2) | -0.04 |
| Peptic ulcer disease | 41084 (29.7) | 11703 (27.4) | 0.05 |
| Cancer | 63955 (46.2) | 14755 (34.6) | 0.24 |
| Falls | 12205 (8.8) | 4725 (11.1) | -0.08 |
| Rheumatoid Arthritis | 3772 (2.7) | 1263 (3.0) | -0.02 |
| Coagulation disorder | 9972 (7.2) | 2894 (6.8) | 0.02 |

**Supplemental Table 6. Patient Characteristics of the study population in the validation cohort**

| **Characteristics** | **Patients with high (top 20%) EHR- continuity**  **N = 95,965** | **Patients with low (lower 80%) EHR- continuity**  **N = 7,096** | **Standardized difference** |
| --- | --- | --- | --- |
| Age, years | 73.74 (7.25) | 75.27 (7.74) | -0.20 |
| Female | 39509 (41.2) | 2325 (32.8) | 0.17 |
| Race |  |  |  |
| White | 78435 (81.7) | 5942 (83.7) | -0.05 |
| Black | 3835 (4.0) | 282 (4.0) | 0.00 |
| Asian | 1285 (1.3) | 56 (0.8) | 0.05 |
| North American Native | 91 (0.1) | 7 (0.1) | 0.00 |
| Unknown | 12319 (12.8) | 809 (11.4) | 0.04 |
| **Medical history** |  |  |  |
| Coronary Artery Disease | 29848 (31.1) | 2506 (35.3) | -0.09 |
| Atrial Fibrillation | 18031 (18.8) | 1465 (20.6) | -0.05 |
| Hypertension | 86373 (90.0) | 6412 (90.4) | -0.01 |
| Heart Failure | 10686 (11.1) | 1019 (14.4) | -0.10 |
| Ischemic Stroke | 6819 (7.1) | 699 (9.9) | -0.10 |
| Acute Myocardial Infarction | 4434 (4.6) | 339 (4.8) | -0.01 |
| COPD | 15279 (15.9) | 1282 (18.1) | -0.06 |
| Pneumonia | 9422 (9.8) | 853 (12.0) | -0.07 |
| Dementia | 8989 (9.4) | 891 (12.6) | -0.10 |
| Depression | 18793 (19.6) | 1500 (21.1) | -0.04 |
| Peptic ulcer disease | 29745 (31.0) | 2099 (29.6) | 0.03 |
| Cancer | 49092 (51.2) | 3274 (46.1) | 0.10 |
| Falls | 8400 (8.8) | 804 (11.3) | -0.08 |
| Rheumatoid Arthritis | 3822 (4.0) | 228 (3.2) | 0.04 |
| Coagulation disorder | 7949 (8.3) | 646 (9.1) | -0.03 |

**Additional method details**

**Cohort Identification algorithm:**

- 1. **Cohort Summary**

1. In the linked Medicare-RPDR dataset, we will identify all the patients aged 65 and above Fee-for-Service (FFS) beneficiaries with Part-D prescription benefits for from 2007-2017, who also have at least 1 encounter recorded in RPDR after 1, January 2007.
   1. **Important steps for cohort formation**
      1. Eligible cohort entry dates

2007/1/1-2017/12/31

- - 1. Specify inclusion/exclusion criteria for cohort entry and define the index date

1. **Cohort eligibility** 🡪 **please count the number of patients that remain at each step**
   - 1. At least 365 days continuous enrollment in claims data (Part A+B+D for Medicare patients and Medicare medical-drug coverage for the Medicaid patients) defined as <32 days enrollment gap using enrollment and disenrollment dates, preceding the index date (one can have up to 31 days gap before the enrollment is considered discontinued.)
     2. At least 1 encounter recorded in RPDR, which overlaps with the continuous enrollment period in (i)
2. Those with different sex in RPDR vs claims will be excluded.
3. Those with DOB in RPDR and claims different >365 days will be excluded**,** if difference<=365, we will use DOB in the claims to calculate age at the index date
4. Censor a patient when 1) loss of enrollment (Part A+B+D for Medicare patients and Medicare medical-drug coverage for the Medicaid patients) 2) death; 3) end of data, whichever comes first.

Note:

Date 1 = # of PT found in the CMS Enrollment A/B/D file.

Date 2 = # of PT have at least 365 days of enrollment (A/B/D).

1. **Continuity measurements**
   1. **Please create the following variables for each person for each year following the index date and overall during follow-up (**Note the time scale is “time since the index date” and we only calculate continuity measures for a complete year, i.e. 365 days, so it will be missing for the terminal year with <365 days. **)**

Steps to calculate continuity measures:

- 1. In CMS data, please exclude all the encounters if Enc_Type=AV if it falls between [admission date+1, discharge date-1] of another inpatient stay for the same patient
  2. In both RPDR and CMS databases, regroup them into the New_Enc_type as follows (excluding those not AV, ED or IP):

| New_Enc_type | Enc_type |
| --- | --- |
| Outpatient | AV or ED |
| Inpatient | IP |

- 1. Match each RPDR encounter to CMS encounter data by admission and discharge date and by “New_Enc_type”, delete the RPDR encounters that cannot be matched in CMS data (delete them only for the purpose of calculating the continuity measures), but keep all the CMS encounters even though you cannot find matches in RPDR.
  2. When calculating the following proportions, please use “New_Enc_type” where New_Enc_type=” Outpatient” are used for the outpatient proportions and New_Enc_type=” Inpatient” are used for the inpatient proportions.

| **Variable name** | **Definition** |
| --- | --- |
| **Enc_Out_RPDR** | $\# Outpatient visits recorded in RPDR$ |
| **Enc_Out_CMS** | $\boldsymbol{\#}Outpatient visits recorded in claims data$ |
| **Enc_In_RPDR** | $\# Inpatient visits recorded in \ldots$ |
| **Enc_In_CMS** | $\# Inpatient visits recorded in claims data$ |
| **Enc_cap** | $\frac{\frac{\# Outpatient visits recorded \ldots EHR}{\# Outpatient visits recorded in claims data}+\frac{\# Inpatient visits record\ldots in EHR}{\# Inpatient visits recorded in claims data}}{2}$ |

Notes:

- - 1. During a hospital stay, a physician consultation from outside may go to carrier files which may be considered an outpatient visit, which will be excluded in step(a) (did not exclude first day and last day overlapping as there could be true outpatient visits on those two days).
    2. Note that if an ED visit results in an admission, it is incorporated with that admission to be one record in the inpatient file in CMS data, but they are likely 2 records in RPDR. In such cases, the RPDR ED encounters will be unmatched in step(c) and be deleted from the calculation.
    3. In the cases of mislabeling of ED vs AV in RPDR (more likely some ED visits get coded as AV as we require clinic_name to be ED but it can be under-coded), if that ED visit did not result in an admission, it will be merged in step(b) and kept in the data in step(c)
    4. As OA are explicitly coded with clinic names in RPDR while they could be imbedded in AV if CMS have pharmacy-only, rad-only, and lab-only visit billed separately, some of the unmatched encounters may simply be due to such mismatches rather than incomplete capture of care across continuum, which will be the same for EHR continuity and discontinuity cohort. Therefore, this measurement can still tell these 2 cohorts apart if they have different match rates otherwise.

**3. K-fold Cross Validation Adjustment for SMOTE**

We implemented Synthetic Minority Oversampling Technique (SMOTE), an oversampling technique to reduce negative influence of an unbalanced dataset^^[[1]](#footnote-1)^^ . Our SMOTE implementation entails adding samples very close, but not identical, to the minority sample so the number of examples in the majority and minority classes are approximately equal. Specifically, the k-nearest neighbors (k = 5 in our case) of a sample in the minority class is chosen and the arithmetic mean of the distance between the sample and each of the k points are computed. The new point is then computed by adding a scaled value (a number between 0 and 1) of the arithmetic mean to the sample. We used a built-in SMOTE implementation in our study (https://imbalanced-learn.org/stable/references/generated/imblearn.over_sampling.SMOTE.html).

**4. Distribution of the follow-up time in the training and validation set (days).**

|  | **Minimum** | **Maximum** | **Median** | **Mean (SD)** |
| --- | --- | --- | --- | --- |
| **Training Set** | 1.0 | 365.0 | 365.0 | 351.77 (58.06) |
| **Validation Set** | 1.0 | 365.0 | 365.0 | 347.86 (65.67) |

1. Wang J, Xu M, Wang H, et al. Classification of Imbalanced Data by Using the SMOTE Algorithm and Locally Linear Embedding. *2006 8th international Conference on Signal Processing* 2006;3:-. [↑](#footnote-ref-1)
