# Supplementary material for: The impact of electronic health record discontinuity on prediction modeling
Source: PLoS One. 2023 Jul 6;18(7):e0287985. doi: 10.1371/journal.pone.0287985 (PMC10325091; doi:10.1371/journal.pone.0287985)
Supplement: Attachment — Submitted filename: Response to the Reviewers_PLOS ONE_FINAL.docx [file pone.0287985.s002.docx]

Introduction to Author Reply:

Dear Editors,

We are grateful for the opportunity to revise our manuscript for consideration in PLOS One. We believe that the queries and suggestions from the Editors and Reviewers have helped to significantly strengthen the manuscript as a whole. To facilitate your review of this manuscript, below please find a point-by-point response to each query, together with the specific changes made in response to each comment (and where those changes can be found within the updated manuscript; **page numbers referred to were that in the marked-up version**). Each comment is listed below, followed by the author response in italics.

Thank you for the opportunity to submit our updated and revised manuscript for consideration.

Sincerely,

Kueiyu Joshua Lin, MD, ScD

**Editor Comments:**

**REVIEWER COMMENTS:**

**Reviewer 1:**

It is not clear from the manuscript if you used EHR data (from Medicare) that was coded or did you somehow extract it from free text. Also, please explain the data in some more detail, including the fields used for the ML algorithms.

*Response: This study utilized data from the Research Patient Data Repository (AMIA Annu Symp Proc 2006:1044), which contains EHR data from two large multi-center delivery networks in Massachusetts. The EHR data were linked to Medicare fee-for-service Parts A (inpatient coverage), B (outpatient coverage), and D (prescription benefits) claims data by insurance policy number, date of birth, and gender (linkage success rate= 98.7%). We did not extract data from the free text notes but have now clarified the data source and how predictors are defined by categories in the 1^st^ and 3^rd^ paragraph under method, respectively.*

**Reviewer 2:**

This paper is well organized and clearly written. The methodology and analysis are comprehensive, and the final conclusion is clearly summarized. The following issues should be addressed:

1. In the methodology, the data processing needs to be clarified: i.e., how to deal with categorical data and numerical data? One-hot vectors or binned numbers? These should be described in the Method or written with Table 1 when presenting the data features.

*Response: we have added information how the predictors were defined and modeled in the 3^rd^ paragraph under Method.*

2. Though AUC scores are given, some insights are missing. For example, what features are causing such AUC difference? The chosen ML models may provide different weights to the input features, it is possible to show such analysis in the Discussion.

*Response: Our experiment set up the comparison to be between those with high vs. low EHR-continuity. The observed AUC difference is thus mainly caused by difference in EHR-continuity. Because the input features were based on the same code-based definitions (see Supplemental Table 1), the difference in model performance is likely due to different levels of misclassification of the input features. For instance, because the feature “Atrial Fibrillation (AF)” is based on having ICD-9 or ICD-10 diagnosis code of AF, this feature is more likely to be misclassified as “not present” for a patient with low EHR-continuity due to AF code recorded outside of the study EHR. Prior studies have shown that a single EHR system captures less than 30% of all the healthcare information, which can translate into up to 17-fold greater misclassification of comparative effectiveness research (CER)-relevant variables in those with a record capture rate<10% vs. ≥80% (Epidemiology 2018;29(3):356-63). When a prediction model is trained in those with low EHR-continuity, the predictors and outcomes may be substantially misclassified. We have expanded our discussion to incorporate these insights and explanation in the 2^nd^ paragraph under Discussion.*

3. It may be possible to add bar charts instead of tables for better results presenting.

*Response: Following the reviewer’s suggestion, we have made the original Table 3 into Figure 1.*

**Reviewer 3:**

This paper applied machine learning algorithms based on EHR data in order to prove that when predicting outcomes like mortality, CV/bleeding events etc., patients with low EHR continuity consistently had worse performance than those who have high EHR continuity. The statement is demonstrated with logical supportive details. There are some problems need to be addressed to offer more clear presentation to readers.

1) From table 2, it seems that either lower or higher 50% of the events only have around 2000 event cases, compared to the cohort like 32K in training, 20K in validation, which means your dataset is extremely imbalanced. It is better to show in details how did you use SMOTE to adjust that imbalance. Also, in the evaluation part, ROC AUC have weakness in showing true performance of imbalanced classification task, it's strongly recommended to add precision recall AUC and show AUC difference between high/low EHR continuity.
*Response: Our* SMOTE implementation *entails adding samples very close, but not identical, to the minority sample so the number of examples in the majority and minority classes are approximately equal. Specifically, the k-nearest neighbors (k = 5 in our case) of a sample in the minority class is chosen and the arithmetic mean of the distance between the sample and each of the k points are computed. The new point is then computed by adding a scaled value (a number between 0 and 1) of the arithmetic mean to the sample. We used a built-in* SMOTE *implementation in our study (https://imbalanced-learn.org/stable/references/generated/imblearn.over_sampling.SMOTE.html). These details have been added to the supplemental materials. We have also now added information on precision-recall AUC to Table 3.*

2)It's not clearly described that how EHR features are formatted in the predictive models. For example, whether dummy variables are generated for categorial predictors? In the medical history, in addition to indicators of diagnosed diseases, whether there are other information can be extracted? Like time interval between diagnosis and events? Also, whether there are treatments or drug use details can be used as predictors?
*Response: we have added information how the predictors were defined and modeled in the 3^rd^ paragraph under Method.*

3)2007 to 2017 is a very wide time range, what time periods are the training and validation cohort data are obtained specifically? Also, it is better to show the patient duration distribution in training/validation like minimal/median/maximum time that a patient can be tracked in the dataset.
*Response: As we are predicting one-year risk of prespecified outcomes, the follow-up was truncated one year after the cohort entry. Below is the distribution of the follow-up time in the training and validation set. We have now added this information in the Appendix under “****Additional method details****.”*

|  | Minimum | Maximum | Median | Mean (SD) |
| --- | --- | --- | --- | --- |
| Train | 1.0 | 365.0 | 365.0 | 351.77 (58.06) |
| Validation | 1.0 | 365.0 | 365.0 | 347.86 (65.67) |

4)More introduction can be added to explain the definition and extent of EHR-continuity.

*Response: Following the reviewer’s recommendation, we have now expanded our discussion (1^st^ paragraph under Introduction).*

**Reviewer 4:**

The authors examine the impact of EHR continuity on the performances of machine learning algorithms to predict three widely investigated clinical outcomes. The authors used an existing model to classify if a patient experienced EHR discontinuity, and the training set was split into two parts, which have either high EHR continuity or low EHR continuity. Then ML models are trained separately using these two parts to report and compare results on an external validation set. The experimental setup of the study is commendable and the statistical analysis is sound, presenting sufficient details to support its conclusion on the impact of EHR continuity on ML models. I have two major comments.

1. EHR continuity was predicted using an off-the-shelf algorithm. I think a bit more detail on the evaluation of this algorithm would better inform the readers. The algorithm can also be viewed as a filter, and we see improved downstream performances after removing the “noisy” half of the training data using the algorithm. This makes me wonder if any other factors might result in the difference. For example, is it possible that 50% of data with low EHR continuity also suffers from a higher level of data missingness? These two are obviously correlated but not equivalent since there could be other causes of data missingness other than EHR discontinuity. Maybe more descriptive statistics on the two datasets with varied EHR continuity would be helpful.

*Response: Our prior study has demonstrated that comparing those with the lowest (<10%) vs. highest (≥80%) level of EHR-continuity, there could be 17-fold greater amount of missing diagnosis or procedure code to define of commonly used CER variables when ascertained only based on EHR (Epidemiology 2018;29(3):356-63). It has also been shown that restricting the analysis to those with top 20% of predicted EHR-continuity can achieve adequate quality of variable classification while preserving representative patient characteristics (Clin Pharmacol Ther 2018;103(5):899-905). We believe that is the primary reasons underlying the difference in model performance (i.e., difference in input feature misclassification). We have now define the EHR-continuity based on this cut-off of top 20% vs. lower 80% predicted EHR-continuity and demonstrate the patient characteristics by EHR-continuity in the training and validation set. This is now included in as Supplemental Table 5 and 6. Note that these added two tables are based on patient characteristics ascertained by the all available data (claims or EHR data), which is not subject to the influence of EHR-discontinuity. We did not observe substantial difference in the comorbidity profile, which has been demonstrated before (Epidemiology 2018;29(3):356-63) and we have discussed that in the 4^th^ paragraph under discussion.*

| **Patient Characteristics of the study population in the training cohort** | | | |
| --- | --- | --- | --- |
| Characteristics | Patients with high (top 20%) EHR- continuity  N = 138,294 | Patients with low (lower 80%) EHR- continuity  N = 42,656 | Standardized difference |
| Age, years | 74.34 (7.68) | 76.05 (8.21) | -0.22 |
| Female | 60129 (43.5) | 17574 (41.2) | 0.05 |
| Race |  |  |  |
| White | 115364 (83.4) | 36023 (84.5) | -0.03 |
| Black | 3383 (2.4) | 1172 (2.7) | -0.02 |
| Asian | 2728 (2.0) | 822 (1.9) | 0.01 |
| North American Native | 101 (0.1) | 51 (0.1) | 0.00 |
| Unknown | 16718 (12.1) | 4588 (10.8) | 0.04 |
| Medical history |  |  |  |
| Coronary Artery Disease | 40045 (29.0) | 13500 (31.6) | -0.06 |
| Atrial Fibrillation | 24226 (17.5) | 8447 (19.8) | -0.06 |
| Hypertension | 123053 (89.0) | 38676 (90.7) | -0.06 |
| Heart Failure | 13230 (9.6) | 4864 (11.4) | -0.06 |
| Ischemic Stroke | 10272 (7.4) | 3348 (7.8) | -0.02 |
| Acute Myocardial Infarction | 4598 (3.3) | 1421 (3.3) | 0.00 |
| COPD | 19580 (14.2) | 6940 (16.3) | -0.06 |
| Pneumonia | 11979 (8.7) | 4583 (10.7) | -0.07 |
| Dementia | 13687 (9.9) | 6573 (15.4) | -0.17 |
| Depression | 27267 (19.7) | 9033 (21.2) | -0.04 |
| Peptic ulcer disease | 41084 (29.7) | 11703 (27.4) | 0.05 |
| Cancer | 63955 (46.2) | 14755 (34.6) | 0.24 |
| Falls | 12205 (8.8) | 4725 (11.1) | -0.08 |
| Rheumatoid Arthritis | 3772 (2.7) | 1263 (3.0) | -0.02 |
| Coagulation disorder | 9972 (7.2) | 2894 (6.8) | 0.02 |

| **Patient Characteristics of the study population in the validation cohort** | | | |
| --- | --- | --- | --- |
| Characteristics | Patients with high (top 20%) EHR- continuity  N = 95,965 | Patients with low (lower 80%) EHR- continuity  N = 7,096 | Standardized difference |
| Age, years | 73.74 (7.25) | 75.27 (7.74) | -0.20 |
| Female | 39509 (41.2) | 2325 (32.8) | 0.17 |
| Race |  |  |  |
| White | 78435 (81.7) | 5942 (83.7) | -0.05 |
| Black | 3835 (4.0) | 282 (4.0) | 0.00 |
| Asian | 1285 (1.3) | 56 (0.8) | 0.05 |
| North American Native | 91 (0.1) | 7 (0.1) | 0.00 |
| Unknown | 12319 (12.8) | 809 (11.4) | 0.04 |
| Medical history |  |  |  |
| Coronary Artery Disease | 29848 (31.1) | 2506 (35.3) | -0.09 |
| Atrial Fibrillation | 18031 (18.8) | 1465 (20.6) | -0.05 |
| Hypertension | 86373 (90.0) | 6412 (90.4) | -0.01 |
| Heart Failure | 10686 (11.1) | 1019 (14.4) | -0.10 |
| Ischemic Stroke | 6819 (7.1) | 699 (9.9) | -0.10 |
| Acute Myocardial Infarction | 4434 (4.6) | 339 (4.8) | -0.01 |
| COPD | 15279 (15.9) | 1282 (18.1) | -0.06 |
| Pneumonia | 9422 (9.8) | 853 (12.0) | -0.07 |
| Dementia | 8989 (9.4) | 891 (12.6) | -0.10 |
| Depression | 18793 (19.6) | 1500 (21.1) | -0.04 |
| Peptic ulcer disease | 29745 (31.0) | 2099 (29.6) | 0.03 |
| Cancer | 49092 (51.2) | 3274 (46.1) | 0.10 |
| Falls | 8400 (8.8) | 804 (11.3) | -0.08 |
| Rheumatoid Arthritis | 3822 (4.0) | 228 (3.2) | 0.04 |
| Coagulation disorder | 7949 (8.3) | 646 (9.1) | -0.03 |

2. The authors developed models using two datasets with varied EHR continuity and evaluated them on the unified validation set. It may be worth considering also splitting the validation set into two parts according to EHR continuity, similar to the development set. Maybe some supplementary results here would better cast light on the impact of EHR continuity and whether the impact is consistent throughout training and testing. Intuitively, both models developed regardless of EHR continuity should perform better on the validation set with higher continuity than on that with lower continuity. But if the model developed using low EHR-continuity data also performs better on data with low rather than high continuity, then maybe distributional shift plays a more significant factor in the different modeling results. In other words, are the development and validation sets equally “continuous”? Would that matter?
*Response: We agree with the reviewer that the validation should be split into high vs. low-continuity cohort, as done in the training set. The results are now done and shown in the revised Table 2.*

One minor comment on data sharing: the authors should specify more explicitly the restrictions preventing them from sharing the data.

*Response: The data that support the findings of this study are available from the Centers for Medicare & Medicaid Services (CMS). Restrictions apply to the availability of these data, which were used under data use agreement with CMS for this study. Data are available from the author(s) with the permission of CMS. Although data cannot be shared with third parties, reasonable requests for re-analysis of the data will be considered by the authors.*
